# Supplementary material for: mimicINT: A workflow for microbe-host protein interaction inference
Source: F1000Res. 2025 Mar 28;14:128. Originally published 2025 Jan 27. [Version 2] doi: 10.12688/f1000research.160063.2 (PMC11982803; doi:10.12688/f1000research.160063.2)
Supplement: Peer review history

# Reviewer report: Reviewer response for version 2

**DOI:** 10.5256/f1000research.179574.r365844

**Published:** 2025-04-10

**Authors:** Leandro Simonetti

**Affiliations:** 1 Uppsala University, Uppsala, Sweden

Choteau et al. article describes "mimicINT", a new open-source software tool aimed at aiding researchers identify human-pathogen interactions at large-scale. The tool can predict domain-domain and domain-motif interactions between a user-provided list of protein sequences from pathogens and human proteins by predicting instances where the pathogen's proteins mimic the human interaction interfaces. MimicINT predicts interactions using experimental data hand curated into the 3did and the ELM databases for domain-domain and domain-motif interactions, respectively. The authors benchmark mimicINT against publicly available host-pathogen interactions experimental data from bacteria and virus, and observe a robust performance correctly identifying these interactions, specially when using mimicINT calculated domain and motif probability scores to filter the output. Overall mimicINT is proven to be a useful tool for exploring the putative interactomes of pathogens with a human host and this can be useful in the current landscape of emerging new diseases.

 Comments:

- In Methods > Implementation, there's a misplaced semicolon: "[...] host protein (iii); the interaction inferences [...]" > "[...] host protein; (iii) the interaction inferences [...]"
- In Methods > Implementation, is not clear to me how the domain annotations gathered in step (ii) are used by mimicINT down the pipeline
- The first section of Results would benefit from having a sub-title, since it's not a general description of the section but a specific set of results
- The output from the Web Server example provides a series of output files to explore. I think it would be useful to include the calculated domain scores and motif probability p-values for the relevant interaction types in case the user wants to use it for filtering the output. It would also be useful to have the predicted motif instance sequence included in the output table.
- Web server was not accessible today (8th of April 2025) because of an expired SSL certificate, so I could not check the description of the downloaded files in the help section though the output folders structure was easy to understand

I found the manuscript to be clearly written, interesting and mimicINT to be a useful tool for the field of host-pathogen interactions since it offers a thorough first look into the putative host hijacked interactome, plus it provides specific interaction regions (domain and or motifs), offering experimentalist targettable interfaces. The manuscript is also very careful to state that the interactions are based on the curated data made available in the databases 3did and ELM, which constitutes a limitation but also shows the importance and usefulness of the effort put into those resources.

Are the conclusions about the tool and its performance adequately supported by the findings presented in the article?

Yes

Is the rationale for developing the new software tool clearly explained?

Yes

Is the description of the software tool technically sound?

Yes

Are sufficient details of the code, methods and analysis (if applicable) provided to allow replication of the software development and its use by others?

Yes

Is sufficient information provided to allow interpretation of the expected output datasets and any results generated using the tool?

Partly

Reviewer Expertise:

Short Linear Motifs, Protein-Protein Interactions, Host-Pathogen Interactions, Bioinformatics

I confirm that I have read this submission and believe that I have an appropriate level of expertise to confirm that it is of an acceptable scientific standard, however I have significant reservations, as outlined above.

# Reviewer report: Reviewer response for version 2

**DOI:** 10.5256/f1000research.179574.r373844

**Published:** 2025-04-09

**Authors:** Ylva Ivarsson

**Affiliations:** 1 Department of Chemistry, Uppsala University, Husargatan, Sweden

The manuscript is fine now with the corrections made. So I recommend that it is accepted.

Are the conclusions about the tool and its performance adequately supported by the findings presented in the article?

Yes

Is the rationale for developing the new software tool clearly explained?

Yes

Is the description of the software tool technically sound?

Yes

Are sufficient details of the code, methods and analysis (if applicable) provided to allow replication of the software development and its use by others?

Yes

Is sufficient information provided to allow interpretation of the expected output datasets and any results generated using the tool?

Yes

Reviewer Expertise:

Expert on the experimental identification and charachterization of SLiM-based interactions.

I confirm that I have read this submission and believe that I have an appropriate level of expertise to confirm that it is of an acceptable scientific standard.

# Reviewer report: Reviewer response for version 1

**DOI:** 10.5256/f1000research.175876.r365838

**Published:** 2025-02-25

**Authors:** Sobia Idrees

**Affiliations:** 1 University of Technology, Sydney, Australia

The manuscript by Choteau et al. introduces mimicINT, a computational workflow designed to facilitate large-scale inference of host-pathogen protein-protein interactions. The workflow focuses on detecting molecular mimicry elements: short linear motifs and host-like globular domains to predict interactions between microbial and human proteins.

 The authors have clearly described the methodology behind mimicINT, making it a valuable resource for studying host-pathogen interactions.

 One of the manuscript's strengths is the application of mimicINT in two use cases: the interaction between Escherichia coli type-3 secreted effectors and human proteins and Marburg virus interactions. These examples demonstrate the biological relevance of the workflow and highlight its potential for uncovering insights into diverse host-pathogen systems.

 The authors have developed an easy-to-use interactive server, making it more straightforward for users to run the workflow. Moreover, the authors have made mimicINT codes available on GitHub, giving access to the workflow and associated code. This will allow others to review and potentially build upon the work, contributing to the tool's transparency and reproducibility.

Are the conclusions about the tool and its performance adequately supported by the findings presented in the article?

Yes

Is the rationale for developing the new software tool clearly explained?

Yes

Is the description of the software tool technically sound?

Yes

Are sufficient details of the code, methods and analysis (if applicable) provided to allow replication of the software development and its use by others?

Yes

Is sufficient information provided to allow interpretation of the expected output datasets and any results generated using the tool?

Yes

Reviewer Expertise:

SLiM mediated interactions.

I confirm that I have read this submission and believe that I have an appropriate level of expertise to confirm that it is of an acceptable scientific standard.

# Response

**Published:** 2025-02-26

**Authors:** Andreas Zanzoni

**Affiliations:** Inserm TAGC UMR_S1090, Aix-Marseille University, Marseille, France

We thank the reviewer for these positive comments.

# Reviewer report: Reviewer response for version 1

**DOI:** 10.5256/f1000research.175876.r363112

**Published:** 2025-02-24

**Authors:** Ylva Ivarsson

**Affiliations:** 1 Department of Chemistry, Uppsala University, Husargatan, Sweden

The manuscript by Choteau et al., describes a useful workflow for bioinformatic analysis of microbe-host protein interactions.

 The manuscript reads well and the method is clearly described. The limitations set by the use of the currently annotated ELM instances is clearly described, and highlights the importance of maintaining such curation efforts.

 As an experimentalist I appreciate in principle the on-line server. However, when testing it, it did not run (“OperationalError at /prediction/run; could not extend file "base/16384/16619_fsm": No space left on device; HINT:  Check free disk space.”) so I could not really evaluate it.  Even when running the text example provided the outcome was the same, which was a bit unfortunate. The authors should ensure that the server is running, or if the issue is on the user side, then please explain the limitations of the usability of the online version to the non-expert.

Are the conclusions about the tool and its performance adequately supported by the findings presented in the article?

Yes

Is the rationale for developing the new software tool clearly explained?

Yes

Is the description of the software tool technically sound?

Yes

Are sufficient details of the code, methods and analysis (if applicable) provided to allow replication of the software development and its use by others?

Yes

Is sufficient information provided to allow interpretation of the expected output datasets and any results generated using the tool?

Yes

Reviewer Expertise:

Expert on the experimental identification and charachterization of SLiM-based interactions.

I confirm that I have read this submission and believe that I have an appropriate level of expertise to confirm that it is of an acceptable scientific standard, however I have significant reservations, as outlined above.

# Response

**Published:** 2025-02-26

**Authors:** Andreas Zanzoni

**Affiliations:** Inserm TAGC UMR_S1090, Aix-Marseille University, Marseille, France

First of all, we thank the reviewer for her positive remarks.

 Regarding the *mimic*INTweb issue raised by the reviewer, we found a miss-configured logging option which filled the server disk space. We have now fixed the problem. This should allow the reviewer to test the webserver.
